# Editorial: Social media neurosurgery and global neurosurgery

**DOI:** 10.3389/fsurg.2024.1480013

**Published:** 2024-10-04

**Authors:** Harsh Deora, Kanwaljeet Garg, Giuseppe Emmanuele Umana, Alfredo Conti, Vishal K. Chavda, Ismail Bozkurt, Bipin Chaurasia

**Affiliations:** ^1^ Department of Neurosurgery, National Institute of Mental Health and Neurosciences, Bengaluru, India; ^2^ Department of Neurosurgery, All India Institute of Medical Sciences, New Delhi, India; ^3^ Department of Neurosurgery, Azienda Ospedaliera Cannizzaro, Catania, Italy; ^4^ Department of Neurosurgery, University of Bologna, Bologna, Italy; ^5^ Department of Medicine, Multispecialty, Trauma and ICCU Centre, Sardar Hospital, Ahmadabad, India; ^6^ Department of Neurosurgery, Medical Park Ankara Hospital, Ankara, Türkiye; ^7^ Department of Neurosurgery, Yuksek Ihtisas University, School of Medicine, Ankara, Türkiye; ^8^ Department of Neurosurgery, Neurosurgery Clinic, Birgunj, Nepal

**Keywords:** social media, neurosurgery, neurosurgery cocktail, global neurosurgery, social media neurosurgery

**Editorial on the Research Topic**
Social media neurosurgery and global neurosurgery

## Introduction

In today's digital world, social media has become a powerful tool in various fields, including neurosurgery. With widespread adoption, social media transforms neurosurgical education, professional development, and global collaboration. This editorial examines the expanding role of social media in neurosurgery, focusing on its impact on education, bridging global disparities, and the ethical challenges it presents.

## The growth of social media among neurosurgeons

As of 2023, over 4.9 billion people—approximately 61.8% of the global population—are active on social media, with the largest neurosurgery social media platform being the “Neurosurgery Cocktail” group (1). Neurosurgeons have increasingly recognized the value of these platforms for professional engagement and knowledge exchange. A 2021 study published in the Journal of Neurosurgery (2) found that 45% of neurosurgeons use social media professionally, signalling a significant shift towards digital interaction. The American Association of Neurological Surgeons (AANS) also reported a 250% increase in social media followers from 2017 to 2022, highlighting the growing importance of these platforms in neurosurgery.

## Revolutionizing neurosurgical education

Social media has revolutionized neurosurgical education by offering a dynamic, real-time knowledge-sharing platform. While effective, traditional educational methods often need more immediacy and global reach than social media provides. Platforms such as Twitter, LinkedIn, and specialized online forums allow neurosurgeons to access the latest research, engage in case discussions, and observe surgical techniques shared by colleagues worldwide. This instant connectivity promotes continuous professional development, ensuring that neurosurgeons stay informed about the latest advancements in their field.

A 2020 survey by the World Federation of Neurosurgical Societies (WFNS) revealed that 78% of neurosurgeons believe social media has significantly enhanced their continuing medical education (3). Additionally, 65% reported using social media to follow academic journals and keep up with developments in neurosurgery. The COVID-19 pandemic accelerated this shift towards digital learning, as many in-person conferences and workshops transitioned to virtual formats. For instance, virtual conferences saw a 300% increase in virtual attendance compared to previous in-person events, demonstrating the accessibility and convenience that social media offers for education (4).

## Addressing global neurosurgical disparities

One of the most significant contributions of social media to global neurosurgery is its ability to connect practitioners across different geographic and economic landscapes. In low- and middle-income countries (LMICs), where access to advanced neurosurgical training and resources is limited, social media is a critical platform for knowledge sharing and mentorship. Recently, a study (5) found that 60% of neurosurgeons in LMICs use social media to seek advice on complex cases and advanced surgical techniques from peers in high-income countries.

Organizations like the WFNS and the Neurosurgery Cocktail have effectively utilized social media to promote global neurosurgical initiatives. These platforms have been pivotal in advancing the goals of the Lancet Commission on Global Surgery, which aims to provide access to safe, affordable surgical care to 80% of the world's population by 2030. Social media campaigns have been instrumental in raising awareness, facilitating fundraising, and advocating for these initiatives, showcasing the power of digital platforms in advancing global neurosurgical care (6–10).

## Ethical considerations in the digital Era

While the benefits of social media in neurosurgery are clear, its use also presents challenges, particularly regarding ethical considerations. The rapid dissemination of information can sometimes lead to the spread of misinformation, which is especially concerning in neurosurgery. Additionally, maintaining patient confidentiality and adhering to ethical standards is crucial. Neurosurgeons may face ethical dilemmas related to social media use, underscoring the need for clear guidelines and education on responsible digital practices.

The article by Ramirez et al. highlights the crucial role that social media platforms play in neurosurgery education, particularly in low and middle-income countries (LMICs). A survey involving 210 medical students, residents, and neurosurgeons from these regions found that most participants use social media, especially WhatsApp and YouTube, for professional purposes like sharing surgical videos, research, and participating in webinars. While social media offers accessible and cost-effective training resources, challenges such as misinformation, digital literacy, and limited internet access remain significant barriers.

A chilling case report by Fu et al. details a severe injury to the inferior vena cava (IVC) during the removal of a pedicle screw in a 31-year-old man who had previously undergone spinal surgery for an L1 compression fracture. Although the initial screw removal was successful, the L2 pedicle screw inadvertently migrated into the retroperitoneum and punctured the IVC, causing a life-threatening situation. A multidisciplinary team successfully repaired the IVC using an artificial graft. The patient recovered well and returned to normal activities after three years. The report emphasizes the risks of vascular injury during pedicle screw removal and the importance of careful surgical techniques.

The article by Chaurasia et al. reviews the evolution and current state of neurosurgery training in Nepal. It highlights the need for uniformity in global neurosurgery training and the specific challenges faced in Nepal, including limited training seats and the necessity for some students to seek education abroad. Despite these challenges, there has been progress, such as increased training seats and the formation of the Nepalese Society of Neurosurgeons (NESON). The article concludes with an optimistic outlook, emphasizing the need for further investment in education and training to meet Nepal's growing healthcare demands.

Unnecessary spine surgery (USS) is a growing concern globally, often resulting from factors like overreliance on MRI findings, financial incentives, and lack of consensus on low back pain management. Despite 80% of spine cases not requiring surgery, unnecessary procedures persist, leading to significant patient risks and increasing healthcare costs. Measures such as establishing musculoskeletal clinics, implementing second-opinion programs, creating clinical pathways, and fostering open discussions on USS are proposed to address this. Surgeons are urged to prioritize professionalism and adhere to ethical guidelines to reduce USS and improve patient outcomes, concerns are beautifully detailed by Alali.

The article by Faisal et al. discusses the educational impact of the Cerebrovascular Q&A webinar series hosted by the Seattle Science Foundation. The study evaluated the series “effectiveness in enhancing global participants” cerebrovascular and endovascular neurosurgery knowledge. The series was well-received, with over 2,000 registrants from 141 countries, particularly for its advanced content and relevance. It also influenced clinical practices and inspired research ideas, highlighting the potential of webinars as a powerful tool for global medical education.

## Conclusion

Social media is reshaping global neurosurgery, offering unprecedented opportunities for education, collaboration, and advocacy. Its profound impact provides a platform for continuous learning, bridging global disparities, and disseminating critical information. However, as the neurosurgical community navigates this digital frontier, it is essential to remain vigilant about ethical challenges and promote responsible social media use. By doing so, social media can continue to play a pivotal role in enhancing neurosurgical practice and improving patient outcomes worldwide.
